# Polyaspartic acid (PASP)-urea and optimised nitrogen management increase the grain nitrogen concentration of rice

**DOI:** 10.1038/s41598-018-36371-7

**Published:** 2019-01-22

**Authors:** Fei Deng, Li Wang, Xiu-Feng Mei, Shu-Xian Li, Shi-Lin Pu, Qiu-Ping Li, Wan-Jun Ren

**Affiliations:** 1 Key Laboratory of Crop Ecophysiology and Farming System in Southwest China of Ministry of Agriculture, Sichuan Agricultural University, Chengdu, China; 2 Institute for New Rural Development, Sichuan Agricultural University, Ya’an, China; 3 Student Affairs, Sichuan Agricultural University, Ya’an, China

## Abstract

Increase in grain nitrogen concentration (GNC), which is directly affected by nitrogen (N) application, can help overcome the issues of malnutrition. Here, the effects of urea type (polyaspartic acid (PASP) urea and conventional urea) and N management method (two splits and four splits) on GNC and N concentration of head rice were investigated in field experiments conducted in Sichuan, China, in 2014 and 2015. N concentration of grain and head rice were significantly (P < 0.05) increased by N redistribution from the leaf lamina, activities of glutamine synthetase (GS), and glutamate synthase (GOGAT) at the heading stage, and N concentration and GOGAT activity in the leaf lamina at the maturity stage. Compared to conventional urea, PASP-urea significantly improved N concentration of grain and head rice by improving the activities of GS and GOGAT, thereby increasing N distribution in the leaf lamina. The four splits method, unlike the two splits method, enhanced N concentration and activities of key N metabolism enzymes of leaf lamina, leading to increased GNC and N concentration in head rice too. Overall, four splits is a feasible method for using PASP-urea and improving GNC.

## Introduction

Currently, approximately one-eighth of the world population is food deprived and more than 10% is chronically malnourished^1^. Crops are one of the basic sources of energy and protein in human and animal diets. Although crop production increased by 0.5–2% annually over the second half of the last century, sustained increase in production and improvement in quality are still required to meet the future needs^1–3^.

Rice is one of the most widely grown cereals in the world and plays a critical role in food security, especially in Asia^4^. However, modern rice production is associated with concerns regarding both yield and quality of grain, as well as the impact of the environment^5,6^. Nutritional quality, which is one of the key components of grain quality, is considered as an essential target for rice development, whereas the improvement in protein concentration of grain is important to improve the nutritional quality^4,7^. Protein is the second most abundant component in rice grain, which provides 29% of the source of daily protein intake for humans in developing countries^4,8^. In China, rice accounts for 41–49% of the protein intake of the population for which rice is the staple food^9^. Nitrogen (N) is one of the fundamental components of proteins, which is crucial to all forms of life, and usually has a key influence on growth and grain yield of crops^10,11^. More than 90% of accumulated N in rice grain is protein N at maturity^12,13^. Therefore, an increase in grain N concentration (GNC) of crops provides a feasible way to combat malnutrition^14^.

Grain N concentration of crops differs with environmental variations, genotype, and agronomic practices^3,14–16^. Of all the agronomic practices, N application is the most direct method to affect GNC of crops. Grain N concentration is associated with N application rate, timing and method of N application, N fertiliser type, as well as synchronisation between N requirement and supply^14,17,18^. Increasing the N application rate increases N uptake; however, the N redistribution efficiency in vegetative organs might be reduced^19–21^. These finally lead to various changes in GNC of crops.

Split N application, a simple and practical N management method, is widely used for increasing crop yield and N use efficiency^22,23^. It has also been confirmed as a feasible method for improving GNC of maize^14^. Polyaspartic acid, a polymer with free carboxylic amide groups, exhibits good adsorbability on cations and anions, is widely used as superabsorbent material and fertiliser synergist^24–26^. PASP-urea is produced by mixing a certain amount of PASP with conventional urea, and is widely used in crop production in China. Previous studies have demonstrated that PASP-urea increases the N balance in paddy fields, leading to improvement in grain yield, N uptake, and N use efficiency of rice, as well as the accumulation of N in the panicle^21,25,27^. However, there exists limited knowledge of the effectiveness of split N application and PASP-urea on GNC of rice.

It is known that both redistribution of N, accumulated in the vegetative organs before the heading stage, and uptake of N, after the heading stage, contribute to GNC of crops^19,20,28^. Mae and Ohira^29^ demonstrated that approximately 80% of the total N in the panicle is contributed by the redistributed N from vegetative organs, especially, from the leaf lamina^28–30^. During the reproductive period, accumulated N in the vegetative organs remobilises to the grains. The reutilisation of N from the senescent organs is controlled by a series of enzymes. NR, GS, and GOGAT are the three key enzymes that regulate the assimilation and recycling of N^28,31,32^. Both application time of N and N fertiliser type have an influence on N metabolism enzymes and N reutilisation^21,33^. However, the relationship between GNC and N redistribution from the leaf lamina using different urea types as fertiliser is not fully understood.

The hypothesis of the present study is that PASP-urea and split N application could improve GNC of rice. Therefore, in this study, a two-year field experiment involving two urea types and two N management methods was conducted in 2014 and 2015. The specific objectives of this study were to (a) investigate the effectiveness of split N application and PASP-urea on N concentration of grain and head rice and N redistribution from the leaf lamina; (b) examine the relationship between GNC and N redistribution characteristics of the leaf lamina; (c) determine the optimal technique for the application of PASP-urea focusing on the improvement of GNC.

## Results

### Analysis of variance

The effects of year, urea type, N management, and their interactions on the activities of NR, GS, and GOGAT in the leaf lamina, N concentration in the leaf lamina, grain, and head rice, and redistributed N from the leaf lamina are shown in Table 1. The effects of year on N concentration of leaf lamina, grain, and head rice, and redistribution of N from the leaf lamina were significant (P < 0.01), mostly due to the variation in weather and soil properties. Urea type and N management method had a significant (P < 0.01) influence on all the tested variables. The interaction between year and N management significantly affected N concentration in the leaf lamina, grain, and head rice, and redistributed N of the leaf lamina, whereas no significant effect of the interaction between year and urea type on redistributed N of the leaf lamina was observed. The interaction between urea type and N management significantly influenced NR activity of the leaf lamina, and N concentration of leaf lamina, grain, and head rice. A significant effect of the interaction between year, urea type, and N management on N concentration of the leaf lamina, grain, and head rice was recorded.

**Table 1:** Analysis of variance for the activities of nitrate reductase (NR), glutamine synthetase (GS), and glutamate synthase (GOGAT) in the leaf lamina, nitrogen concentration of leaf lamina, grain, and head rice, and redistributed nitrogen from the leaf lamina in response to year, urea type, N management, and their interactions.

Source	Nitrate reductase activity	Glutamine synthetase activity	Glutamate synthase activity	Nitrogen concentration in the leaf lamina	Redistributed nitrogen in the leaf lamina	Grain nitrogen concentration	Head rice Nitrogen concentration
Year	—	—	—	**	**	**	**
Urea type	**	**	**	**	**	**	**
N management	**	**	**	**	**	**	**
Y × U	—	—	—	*	NS	**	**
Y × N	—	—	—	**	**	**	**
U × N	**	NS	NS	**	NS	**	**
Y × U × N	—	—	—	**	NS	**	**

*** and NS indicate significance at *P* ≤ 0.05, *P* ≤ 0.01, and no significance, respectively.

### Activities of nitrate reductase, glutamine synthetase, and glutamate synthase

**Figure 1:**
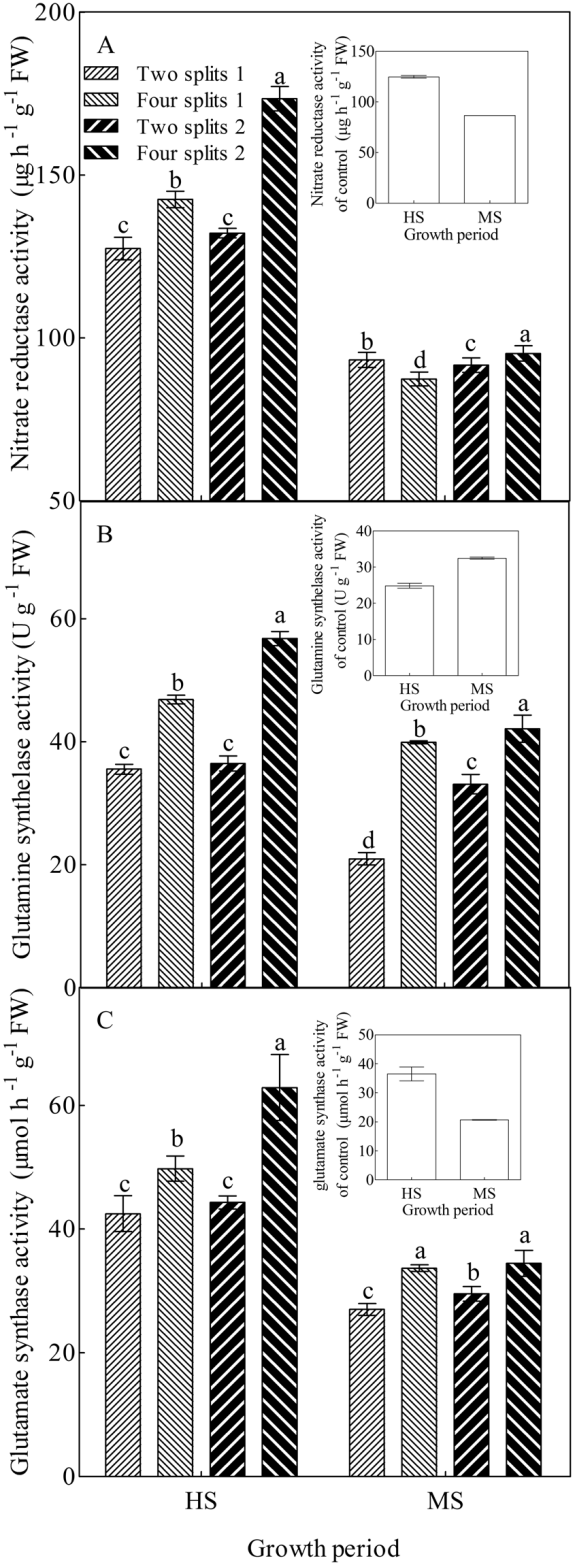
Activities of nitrate reductase (**A**), glutamine synthetase (**B**), and glutamate synthase (**C**) in the leaf lamina in response to urea type and N management in 2014. Different lowercase letters within a group of treatment represent significant (P < 0.05) differences among treatments. The control was not included in the analysis of variance and multiple comparisons. The bar with each column indicates the range of the standard error (SE) of the mean. HS, heading stage; MS, maturity stage.

Except for GS activity of CK, the activities of NR, GS, and GOGAT in all the treatments were found to decrease from the heading stage to the maturity stage (Fig. 1). With the exception of GS activity in two splits1 and two splits2 and NR activity in four splits1 at the maturity stag, N application improved the activities of NR, GS, and GOGAT at both the stages. Compared to the treatment with conventional urea, treatments with PASP-urea increased the activities of NR, GS, and GOGAT by 13.2%, 13.1%, and 16.1%, respectively, at the heading stage, and by 3.4%, 23.5%, and 5.4%, respectively, at the maturity stage. Compared to two splits, four splits significantly improved the activity of NR at the heading stage, as well as the activities of GS and GOGAT at both the stages. Among the four N treatments, the activity of NR in the heading stage, and activities of GS and GOGAT at both the stages were in the order, four splits2> four splits1> two splits2> two splits1. The use of PASP-urea with four splits obviously enhanced the activities of NR, GS, and GOGAT in the leaf lamina, indicating a higher N metabolism in four splits2.

### Nitrogen concentration and redistribution from the leaf lamina

**Figure 2:**
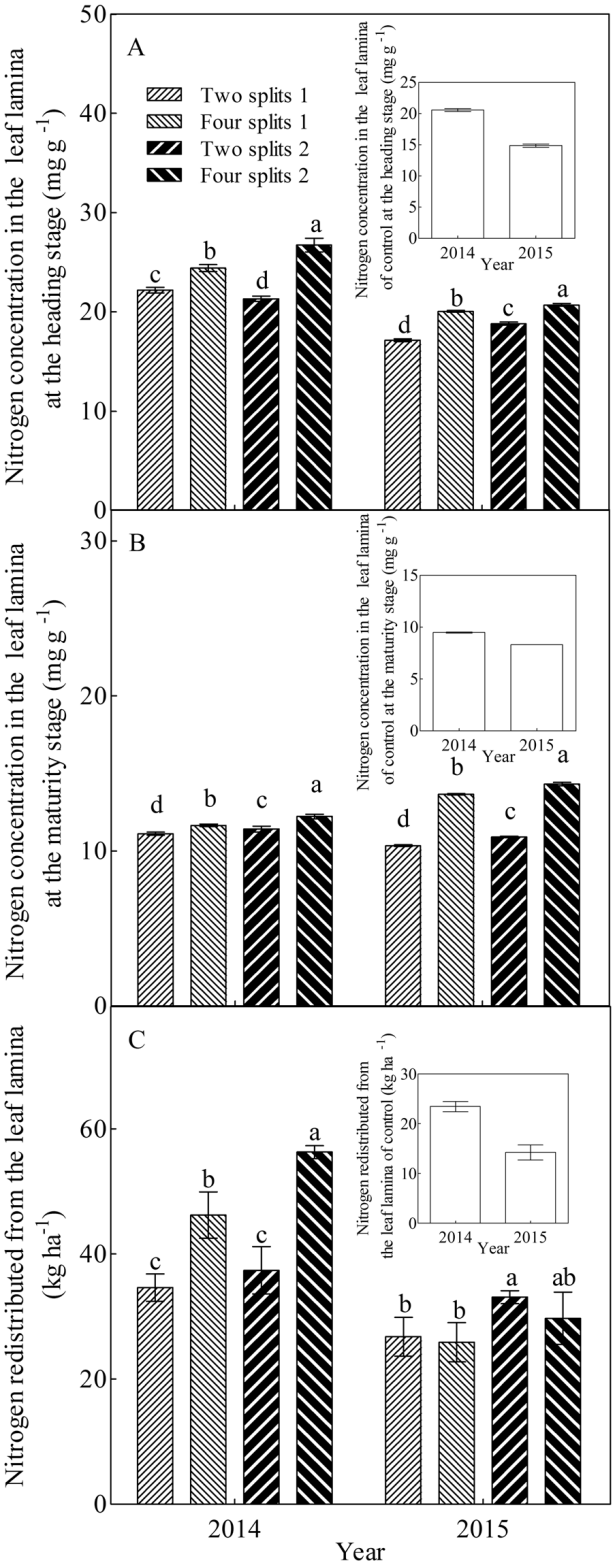
Nitrogen concentration in the leaf lamina at the heading stage (**A**) and maturity stage (**B**), and the nitrogen redistributed from the leaf lamina (**C**) in response to year, urea type, and N management. Different lowercase letters within a group of treatment represent significant (P < 0.05) differences among treatments. The control was not included in the analysis of variance and multiple comparisons. The bar with each column indicates the range of the standard error (SE) of the mean.

Nitrogen concentration and redistribution in the leaf lamina varied with year, urea type, and N management (Fig. 2). N application significantly increased N concentration and redistribution in the leaf lamina. No significant difference in N concentration in the leaf lamina at the maturity stage was observed between 2014 and 2015 (Fig. 2B). However, N concentration in the leaf lamina at the heading stage was remarkably higher in 2014, which contributed to a significant enrichment in N redistribution from the leaf lamina (Fig. 2A,C). Furthermore, N concentration and redistribution in the leaf lamina were significantly improved by PASP–urea and four splits. With the interaction between year and urea type, N concentrations in the leaf lamina at the heading and maturity stages were increased by PASP-urea-2014 and PASP-urea-2015, respectively. Nitrogen concentration in the leaf lamina at the heading stage and N redistribution from the leaf lamina were improved by four splits-2014, whereas N concentration in the leaf lamina at the maturity stage was increased by four splits-2015. Furthermore, both N concentration and redistribution from the leaf lamina were in the order, four splits2 > four splits1 > two splits 2 > two splits1, and four splits2-2014 and four splits2-2015 possessed the highest N concentration in the leaf lamina at the heading and maturity stages, respectively. These results suggest the potential of using PASP-urea with the four splits method for improving N redistribution from the leaf lamina.

### Nitrogen concentration of grain and head rice

**Figure 3:**
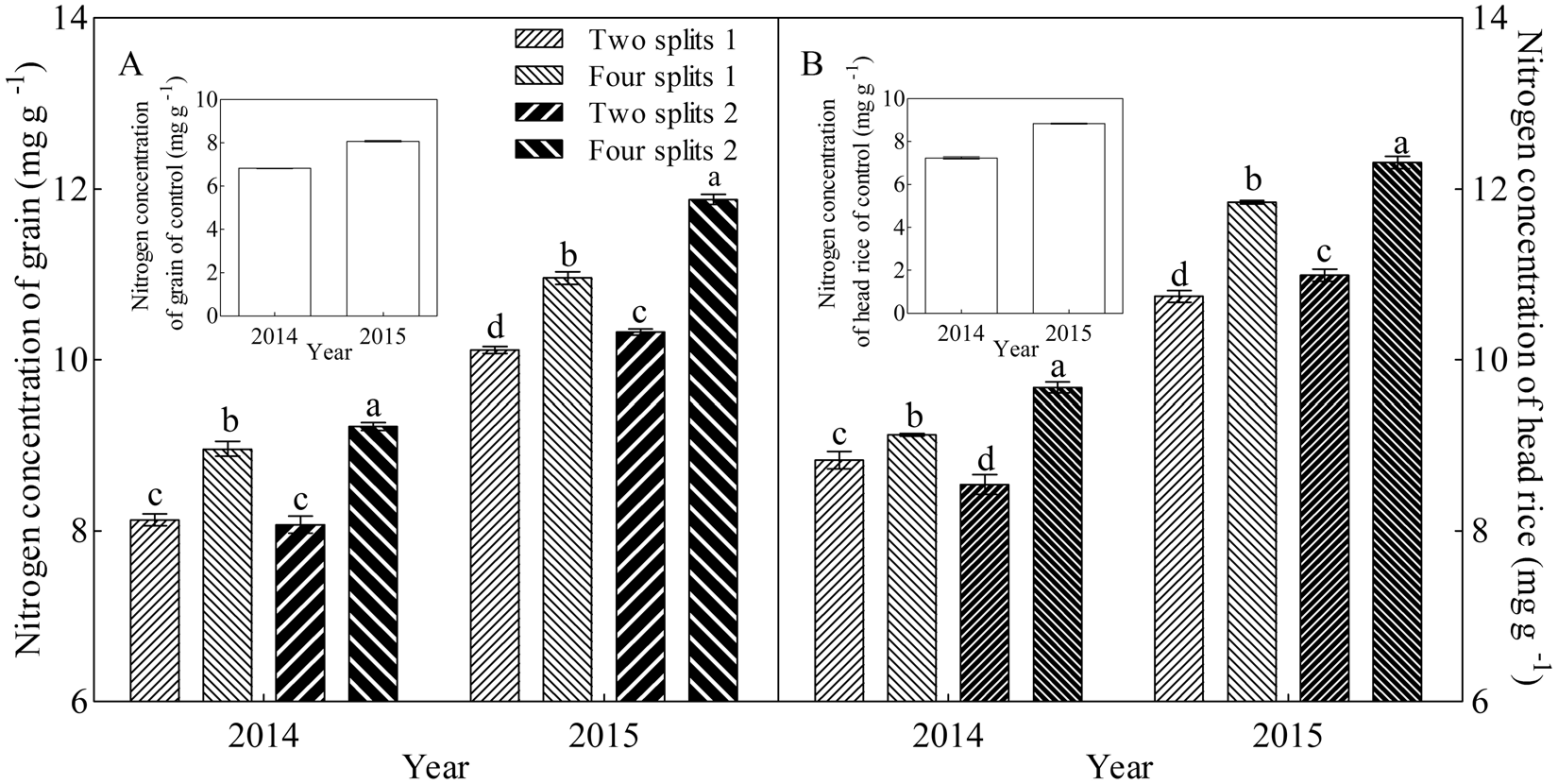
Nitrogen concentration (**A**) and accumulation (**B**) in grain in response to urea type and N management in 2014 and 2015. Different lowercase letters within a group of treatment represent significant (P < 0.05) differences among treatments. The control was not included in the analysis of variance and multiple comparisons. The bar with each column indicates the range of the standard error (SE) of the mean.

N application significantly increased both GNC and N concentrations of head rice (Fig. 3). Compared to those in 2014, GNC and N concentrations of head rice were increased in 2015. Compared to conventional urea and two splits, PASP-urea and four splits significantly improved N concentration of grain and head rice. In 2015, with two splits, both GNC and N concentration of head rice were higher with PASP-urea compared with conventional urea, in contrast to 2014, in which there was no increase in GNC and a decrease in N concentration of head rice with PASP-urea when there were only two splits. With the interaction between year and N management, the effect of four splits compared with two splits was greater in 2015, at 11.3% increase in N concentration of head rice, compared with 2014, at 8.1% increase. Moreover, using PASP-urea with four splits significantly increased N concentration of grain and head rice. Compared to two splits and four splits1, splits2-2014 and four splits2-2015 led to 2.9–14.3% and 8.4–15.0% increases in GNC and 6.0–13.2% and 3.9–14.6% increases in N concentration of head rice were, respectively.

### Relationships between grain nitrogen and leaf lamina nitrogen

The relationships between N concentration of grain and head rice with leaf lamina N concentration, redistribution, and key enzymes activities of nitrogen metabolism are shown in Table 2. Both GNC and N concentration of head rice were significantly (P < 0.01) improved with increased N concentration in the leaf lamina at the maturity stage and redistributed N from the leaf lamina in both the years. Similar results were recorded between N concentration of grain and head rice with N concentration in the leaf lamina at the heading stage in 2014. The N concentration of grain and head rice also significantly (P < 0.01) increased with enhanced activity of GOGAT at both the stages, as well as with the activity of GS at the heading stage in 2014.

**Table 2:** Correlation coefficients (r) between the nitrogen concentration of grain and head rice with the activities of nitrate reductase (NR), glutamine synthetase (GS), and glutamate synthase (GOGAT) in the leaf lamina, nitrogen concentration in the leaf lamina, and redistributed nitrogen from the leaf lamina.

Index	Nitrogen concentration of grain	Nitrogen concentration of head rice
**2014**		
Nitrogen concentration in the leaf lamina at the heading stage	0.890^*^	0.947**
Nitrogen concentration in the leaf lamina at the maturity stage	0.969^**^	0.882*
Redistributed nitrogen from the leaf lamina	0.964^**^	0.971**
Nitrate reductase activity in the leaf lamina at the heading stage	0.783	0.786
Nitrate reductase activity in the leaf lamina at the maturity stage	0.514	0.686
Glutamine synthetase activity in the leaf lamina at the heading stage	0.960^**^	0.945**
Glutamine synthetase activity in the leaf lamina at the maturity stage	0.528	0.382
Glutamate synthase activity in the leaf lamina at the heading stage	0.885^*^	0.890*
Glutamate synthase activity in the leaf lamina at the maturity stage	0.987^**^	0.939**
**2015**		
Nitrogen concentration in the leaf lamina at the heading stage	0.788	0.789
Nitrogen concentration in the leaf lamina at the maturity stage	0.966^**^	0.979**
Redistributed nitrogen from the leaf lamina	0.949^**^	0.959**

^*^Significant at the 0.05 probability level;^**^Significant at the 0.01 probability level.

## Discussion

### Effect of PASP-urea and N management method on N characteristics of the leaf lamina

Application of N fertilisers is a common practice in field crop management. However, the effect of N fertilisers on crops is influenced by fertiliser type, N rate, and application time^14,19,22^. PASP-urea, one of the high efficiency fertiliser, is demonstrated to increase grain yield and N use efficiency of crops^21,24^. By using PASP-urea, better synchronisation between N requirement and supply was realised, which contributed to an increase in photosynthetic capacity, dry matter accumulation, as well as effective panicles, grain filling percentage, and grain weight, and thereby significantly improved the grain yield^21,25,27^. It is well demonstrated that increasing the N rate and optimising the timing of N supply usually result in higher N concentration and accumulation in the rice organs^34,35^. Because of the improved balance between N requirement and supply in the paddy field caused by PASP-urea and four splits^21^, a significant improvement in N concentration of the leaf lamina was observed in the present study (Fig. 2). Furthermore, PASP-urea also significantly increased N redistribution from the leaf lamina. Thus, the use of PASP-urea with the four splits method might be a feasible way to enhance N redistribution from the leaf lamina.

Nitrate reductase is the first rate-limiting enzyme that controls nitrate assimilation in the leaf lamina of most crops^31,36^. In addition, the GS/GOGAT cycle, discovered by Lea and Miflin^37^, is the only route for primary assimilation of ammonium in plants and the essential step for N recycling in senescent vegetative organs^28,30,32^. Many studies have demonstrated the effect of N application on N metabolism in rice^36,38–40^. In the present study, both PASP-urea and four splits could enhance N metabolism in the leaf lamina. Similar to the results of Sun *et al*.^33^, four splits significantly improved the GS and GOGAT activities in the leaf lamina at the heading and maturity stages, as well as NR activity in the leaf lamina at the heading stage (Fig. 1). In addition, PASP-urea also significantly increased the activities of NR, GS, and GOGAT in the leaf lamina, which contributed to the obvious increase in N concentration and N redistribution in the leaf lamina (Figs 1 and 2).

### Effect of PASP-urea and N management method on N concentration of grain and head rice

The GNC of crops has an important nutritional value, because crop grains or seeds are the primary source of proteins in the diet of humans and animals^8,41,42^. Application of N fertiliser is one of the major field management practices that play an important regulatory role on the concentrations of GNC or protein in crops^14,18,43^. In the present study, four splits significantly increased GNC of grain and N concentration of head rice (Fig. 3). This is in agreement with the results of Ning *et al*.^7^, which indicate that N fertiliser has a remarkable effect on protein concentration in rice grain, whereas topdressing N has a larger influence. Furthermore, PASP-urea also had a positive effect on N concentration of grain and head rice. These results demonstrated that the hypotheses of the present study were correct.

It has been clearly demonstrated that approximately 80% of N in the panicle is remobilised through the phloem from senescing organs^28–30^. Indeed, the leaf lamina is considered to be the main source of assimilates and N for grain filling of rice^44^. Approximately half of the accumulated N in the panicle is contributed by the leaf lamina^21^. In the present study, significant positive relationships were observed between N concentration of grain and head rice with N concentration and redistribution of the leaf lamina (Table 2). Furthermore, both N concentration of grain and head rice were increased with enhanced activities of GOGAT and GS (Table 2). This corroborates the findings of Tabuchi *et al*.^28^ and Tamura *et al*.^30^, who suggested that since the major form of N in the phloem sap is Gln, synthesis of Gln controlled by the GS/GOGAT cycle is the essential step for the reutilisation of N in senescing organs. During the senescence progress of the leaf lamina, both PASP-urea and four splits could increase the activities of GS and GOGAT in the leaf lamina at the heading and maturity stages; this promoted the remobilisation of N from the leaf lamina, and contributed to the significant increase in GNC and N concentrations of head rice (Figs 1–3). Therefore, using PASP-urea together with the four splits method is a feasible way to improve N concentration of grain and head rice.

## Conclusions

The increase in crop yield and GNC provides a promising strategy to overcome hunger and malnutrition. Rational N application is a feasible method for improving the N concentration and protein in grains. The present study investigated the effectiveness of PASP-urea and N management method on N concentration and accumulation in rice grain, and their relationships with the N characteristics of the leaf lamina. Taken together, our findings support the strategy for increasing GNC of rice by using PASP-urea. N concentration of grain and head rice, and N redistribution characteristics of the leaf lamina were significantly influenced by urea type and N management method. Both PASP-urea and split N application could improve GNC of rice. With the use of PASP-urea, the activities of NR, GS, and GOGAT, and N concentration and redistribution of leaf lamina were increased, contributing to the improvement in N concentration of grain and head rice. Overall, using PASP-urea with four splits is a feasible method for increasing GNC and N concentration of head rice.

## Materials and Methods

### Study site and materials

The field experiment was conducted at the same field of Huihe farm of Sichuan Agricultural University in Wenjiang (30°43′N, 103°52′E), Sichuan, China, in 2014 and 2015. The previous crop in both years was wheat. Wenjiang had a subtropical humid monsoon climate, with an average air temperature and precipitation of 23.9 °C and 715.0 mm, respectively, during the period from transplantation to harvest (May to September) in 2014, and 24.0 °C and 568 mm, respectively, in 2015^25^. The properties of the top soil layer (0–30 cm) are shown in Table 3. Fyou-498, a mid-late indica hybrid rice variety, bred by Sichuan Agricultural University, was used in the study. Conventional urea and PASP-urea with 46% N were supplied by Sichuan Meiqing Cyanamide Co., Ltd., China.

**Table 3:** Properties of the top soil layer (0–30 cm) in the experimental field in 2014 and 2015.

Year	Soil texture	pH	Organic matter (g kg^−1^)	Total N (g kg^−1^)	Alkali hydrolysable N (mg kg^−1^)	Olsen-P (mg kg^−1^)	Exchangeable K (mg kg^−1^)
2014	medium loam	5.1	37.6	2.2	130.0	12.2	119.5
2015	medium loam	5.2	28.9	1.9	135.5	19.8	105.7

### Experimental design

A two-factor, randomised block experiment, with three replicates, was conducted in 2014 and 2015. The complete experimental details were described previously by Deng *et al*.^25,27^. In brief, the treatments were as follows: (1) two urea types, conventional urea and PASP-urea were used; (2) two N management methods, two splits N application (180 kg N ha^−1^, by which 70% and 30% of N were applied at the basal and tillering stages, respectively) and four splits N application (180 kg N ha^−1^, split-applied as 35% at the basal, 15% at the tillering, 30% at the panicle initiation, and 20% at the spikelet differentiation stages); zero-N treatment (CK) with three replicates was set as the control. The management methods using conventional urea were designated as 1 (e.g. two splits1 and four splits1), whereas those using PASP-urea were designated as 2 (e.g. two splits2 and four splits2).

In both the years, 0.2-m-wide low banks, covered with a plastic film inserted at a depth of 0.3 m in the soil, were used to prevent leakage of fertiliser to the adjacent plots. The dimension of each plot was 10 × 3 m and 9 × 3 m in 2014 and 2015, respectively. In all the treatments, 90 kg·ha^−1^ of P_2_O_5_ (as a single superphosphate) was applied at the basal stage, and 180 kg·ha^−1^ of K_2_O (as potassium chloride) was equally divided at the basal and panicle initiation stages. Seedlings were raised in upland nursery conditions, with 75 kg N ha^−1^ as seed fertilizer. On May 24, 2014, and May 31, 2015, 35-day-old seedlings were transplanted at 26.7 cm × 16.7 cm spacing. Standard chemicals, such as avermectin, validamycin, cyhalofop-butyl were used to control insects (*Chilo suppressalis*), pathogens (false smut), and weeds (barnyard grass) to avoid yield losses. Water management was conducted using a high-efficiency irrigation technique: damp irrigation before the panicle initiation stage, saturated irrigation from the panicle initiation to heading, and alternate wetting and drying irrigation after the heading stage^21,25^. Each plot was irrigated before fertiliser application.

### Redistribution of N from the leaf lamina and N concentration of grain and head rice

At the heading and maturity stages, five representative hills were sampled from the middle of each plot. The leaf lamina from each sample was oven-dried at 105 °C for 1 h and then at 80 °C until obtaining a constant weight. On September 11, 2014, and September 15, 2015, rice plants from each plot were hand-harvested. Approximately 2 kg of hand-harvested grains were sampled. Grains were stored under ventilated conditions for 3 months, and then they were divided into two parts, one part was used to determine the GNC, and another part was shelled and milled to produce head rice. Samples were crushed and sifted through a 0.5-mm screen, and then the N concentration of the leaf lamina, grain, and head rice was measured by the Kjeldahl method. Redistribution of N from the leaf lamina was calculated as the difference in N weight between the heading and maturity stages^21^.

### Activities of nitrate reductase, glutamine synthetase, and glutamate synthase in the leaf lamina

Ten flag leaves were sampled from each plot at the heading and maturity stages, in 2014. The midrib of the leaf was removed and its lamina was sliced and frozen in liquid N, and kept at −80 °C, until further analysis. Nitrate reductase, GS, and GOGAT activities in the flag leaves were determined using commercial chemical assay kits (Jiangsu Keming Biotechnology Institute, Suzhou, China). For the measurement of NR activity, frozen leaf tissues were soaked in inducing medium for 2 h, and then blotted moist tissues were stored at -20 °C for 20 min. Approximately 0.1 g (fresh weight) leaf tissue was homogenised in a triturator with 1.0 mL prechilled extraction medium and then centrifuged at 4000 × *g* for 10 min at 4 °C. The supernatant was used for the NR activity analysis according to the manufacturer’s instructions. The activity of NR (g h^−1^ g^−1^ FW) was determined as the production of NaNO_2_ per hour per gram fresh sample. For the measurement of GS and GOGAT activities, approximately 0.1 g leaf tissues was homogenised with 1.0 mL prechilled extraction medium and centrifuged at 8000 × *g* for 10 min at 4 °C, and then the supernatant was used for the analysis of GS and GOGAT activities according to the manufacturer’s instructions. The activity of GS (U g^−1^ FW) was defined as the quantity of 0.01 variation in the absorbance at 540 nm per minute per gram fresh sample. The activity of GOGAT (μmol h^−1^ g^−1^ FW) was described as the micromole of NADH oxidised per hour per gram fresh sample.

### Data analysis

Analysis of variance of all the data, except the control, was performed using the general linear model of SPSS version 18.0 (IBM, Inc., Chicago, IL, USA). Nitrogen concentration in the leaf lamina and activities of NR, GS, and GOGAT were analysed using the repeated measures analysis model. For the analysis, year, urea type, N management, and sampling time were considered as the fixed effects, and the replicates were considered as the random effects. The LSD test (P < 0.05) was used to determine the differences among the treatments.

## Data Availability

No datasets were generated or analysed during the current study.
